# Misdiagnosis of Babesiosis as Malaria, Equatorial Guinea, 2014

**DOI:** 10.3201/eid2408.180180

**Published:** 2018-08

**Authors:** Marta Arsuaga, Luis Miguel González, Enrique Salvador Padial, Arigecho Woubshet Dinkessa, Elena Sevilla, Elena Trigo, Sabino Puente, Jeremy Gray, Estrella Montero

**Affiliations:** Hospital Universitario La Paz-Carlos III, Madrid, Spain (M. Arsuaga, E. Trigo, S. Puente);; Centro Nacional de Microbiología, Madrid (L.M. Gonzalez, E. Sevilla, E. Montero);; La Paz Medical Center, Bata, Equatorial Guinea (E.S. Padial, A.W. Dinkessa);; University College Dublin, Dublin, Ireland (J. Gray)

**Keywords:** *Babesia microti*, Equatorial Guinea, human babesiosis, malaria, parasites, vector-borne infections

## Abstract

We report a case of babesiosis, caused by *Babesia microti*, in a missionary who worked in Equatorial Guinea but also visited rural Spain. The initial diagnosis, based on clinical features and microscopy, was malaria. The patient’s recovery was delayed until she received appropriate treatment for babesiosis.

*Babesia* parasites are naturally transmitted by ixodid ticks; the parasites invade erythrocytes, causing babesiosis in animals and humans. The disease can be clinically silent or can progress to a fulminant malaria-like disease. Of the 4 characterized *Babesia* species involved, *B. microti* is the one that mostly infects humans and is found worldwide; most cases occur in the United States (*1*). Babesiosis in humans in Africa has rarely been reported (*2**–**4*), but the similarity to malaria, in symptoms and appearance in blood smears, may confuse diagnosis and result in underreporting (*5**,**6*).

In March 2014, a 43-year-old woman with fever, chills, fatigue, and general malaise was admitted to the General Hospital of Douala, Douala, Cameroon. Giemsa-stained blood smears showed intraerythrocytic parasites, leading to a diagnosis of malaria. The patient, who had previously had malaria, was given dyhydroartemisin plus primaquine, improved slightly, and was discharged. A few days later, she was admitted to the Hospital La Paz in Bata, Equatorial Guinea, with similar symptoms. Over an 8-month period, she received 6 consecutive diagnoses of malaria; treatment with quinine, artemether, atovaquone/proguanil, or artemether/lumefantrine led to no clear improvement. Because all antimalarial therapies had failed, the patient’s case was reevaluated.

Chest radiographs and abdominal ultrasonograms were unremarkable. The patient had an intact spleen of normal size and had not received any blood products. Laboratory findings were unremarkable except for a high proportion of neutrophils (86%) (Table). New Giemsa-stained thin blood smears were examined and, in addition to ring forms, rare tetrads (Maltese crosses, which do not occur in *Plasmodium* infections) were observed. A diagnosis of babesiosis with a parasitemia of >0.5% was determined, and the patient then received oral azithromycin (500 mg/d) and atovaquone/proguanil (250 mg/100 mg every 8 h). Untreated blood was sent to the National Center for Microbiology and Hospital La Paz, both in Spain, where *Babesia* spp., but not *Plasmodium* spp., were detected by PCR (Technical Appendix**)**. The partial amplified product was cloned by using TOPO TA vector (ThermoFisher Scientific, Inc., Carlsbad, CA, USA) and sequenced. The short nucleotide sequence of a 157-bp fragment of the *B. microti* 18S RNA gene (online Technical Appendix) showed 100% identity with the *B. microti* Munich type (GenBank accession nos. AB366158, AY789075, AB071177, KT271759, and KX758442). However, precise identification of the strain of parasite involved would have required larger fragments of the 18S rRNA gene. Attempts to amplify the β-tubulin gene were not successful.

**Table:** Initial laboratory test results from blood samples of a patient with suspected babesiosis, Equatorial Guinea, 2014

Test	Result (reference range)
Hematology	
Leukocytes, × 10^3^ μL	10.29 (3.0–10.0)
Hemoglobin, g/dL	13.2 (12.0–16.2)
Mean corpuscular volume, fL	85.9 (79–93)
Hematocrit, %	40.3 (33–44)
Platelet count, × 10^3^ μL	205 (120–400)
Neutrophils, %	86.7 (49–70)
Lymphocytes, %	8.6 (20–50)
Monocytes, %	4.3 (4–8)
Eosinophils, %	0.4 (0–6)

Biochemistry	
Aspartate aminotransferase, U/L	15 (15–46L)
Alanine aminotransferase, U/L	20 (13–69)
Gamma-glutamyl transferase, U/L	14 (12–56)
Lactate dehydrogenase, U/L	450 (300–618)
Alkaline phosphatase, U/L	58 (30–130)
Total protein, g/dL	6.9 (6.3–10)
Albumin, g/dL	4.3 (3.5–5.5)
Bilirubin, mg/dL	0.7 (0.3–1.3)
Indirect bilirubin, mg/dL	0.7 (0.2–0.9)

One week after diagnosis and commencement of specific treatment, the patient traveled to Spain and was admitted to the Unit for Tropical Diseases at the tertiary Hospital La Paz in Madrid, where diagnostic tests for *Babesia* spp., *Plasmodium* spp., and other pathogens were conducted. An indirect immunofluorescence assay (IFA) (Fuller Laboratories, Fullerton, CA, USA) obtained *B. microti* titers of 128. Not surprisingly, anti–*Plasmodium falciparum* antibodies were also detected (titer 640) by IFA by a *Falciparum*-Spot IF kit (bioMérieux S.A., Lyon, France). Diagnostic test results were negative for *Schistosoma* spp., *Strongyloides stercoralis*, *Trypanosoma brucei*, *Leishmania*, *Borrelia*, *Anaplasma*, filariae, *Treponema pallidum*, hepatitis viruses (A, B, and C), HIV, and dengue virus. Treatment for babesiosis was continued for another 14 days, after which PCR results were negative and the patient’s general condition had clearly improved. One year later, PCRs indicated that she was still free of *Babesia* parasites.

We do not have solid evidence of the source of this patient’s babesiosis. Every year since 2001, she spent a week in rural areas in Spain, where at least 1 case of human babesiosis caused by *B. microti* (also “Munich” type) has been recorded (*7*). However, initial symptoms occurred while the patient was in Equatorial Guinea, having arrived there several months earlier from Valencia, Spain, where the *B. microti* vectors in Europe, *Ixodes ricinus* ticks, are not known to occur. However, no data are available on the presence of *Ixode*s ticks or of vertebrate reservoirs infected with *B. microti* in Equatorial Guinea. This lack of information, together with the fact that the patient traveled to different locations inside and outside Africa, makes it difficult to determine whether the infection was acquired in Equatorial Guinea. In such regions, where infrastructure and resources are limited, molecular and serologic diagnostic methods are usually lacking, and diagnoses of febrile diseases are based on symptoms, physical findings at examination, and microscopy. These limitations, and the similarities between malaria and babesiosis, are sufficient to explain why this patient’s babesiosis was initially misdiagnosed as malaria. Because of this misdiagnosis, the patient was treated for malaria 6 times over 8 months. An accurate diagnosis and appropriate treatment for babesiosis was necessary to end this sequence of mistakes. Increased awareness of the possibility of babesiosis, together with appropriate diagnosis, may result in the discovery of more cases of babesiosis in malaria-endemic areas.

Technical Appendix Results of seminested PCR and DNA sequencing for *Babesia microti*.
