# Supplementary material for: Misdiagnosis of Babesiosis as Malaria, Equatorial Guinea, 2014
Source: Emerg Infect Dis. 2018 Aug;24(8):1588–9. doi: 10.3201/eid2408.180180 (PMC6056097; doi:10.3201/eid2408.180180)
Supplement: Technical Appendix — Results of seminested PCR and DNA sequencing for Babesia microti. [file 18-0180-Techapp-s1.pdf]

Article DOI: https://doi.org/10.3201/eid2408.180180

Babesiosis Misdiagnosed as Malaria, Equatorial Guinea, 2014

Technical Appendix

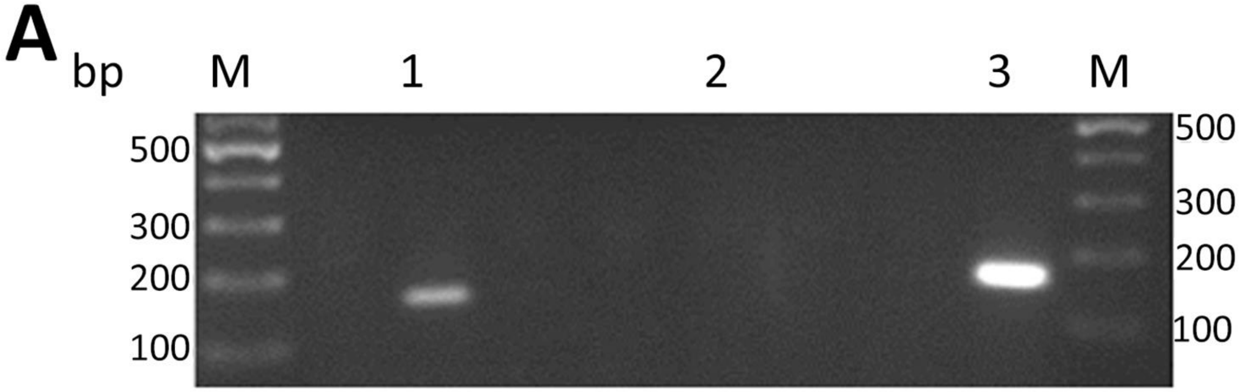

B

```
B. microti 5´ GTTATAGTTTATTTGATGTTCGTTTTACATGGATAACCGTGGTAATTCTAGGG   53
              CTAATACATGCTCGAGGCGCGTTTTACGCGTGGCGTTTATTAGACTTTAACCA  106
              GCCCCTTTGGGTAATCGGTGATTCATAATAAATTAGCGAATCGCATGGCTT 3´ 157
```

Technical Appendix Figure. *Babesia microti* confirmation by seminested-PCR and DNA sequencing. A) Seminested-PCR carried out using the patient peripheral blood yielded a single partial 157-bp 18S rRNA gene fragment. DNA from *B. microti* Gray (ATCC 30221) was used as a positive control. Lane M, size marker; lane 1, amplification from the patient blood; lane 2, negative control; lane 3, amplification from the DNA control. B) 157-bp nucleotide sequence of the partial 18S rRNA gene fragment of *B. microti*. Residue numbering for the sequence is shown on the right.
